# Supplementary material for: A UMLS-based spell checker for natural language processing in vaccine safety
Source: BMC Med Inform Decis Mak. 2007 Feb 12;7:3. doi: 10.1186/1472-6947-7-3 (PMC1805499; doi:10.1186/1472-6947-7-3)
Supplement: Additional file 13 — RAP application directory. Sets up directory for RAP (RDF application for PHP) source code [file 1472-6947-7-3-S13.gz › rap/doc/phpdoc/__filesource/fsource_default__vocabularyRDF_C.php.html]

xml version="1.0" encoding="iso-8859-1"?


File Source for RDF\_C.php


# Source for file RDF\_C.php

Documentation is available at RDF\_C.php

```
1. <?PHP
2. // ----------------------------------------------------------------------------------
3. // Resource Description Framework (RDF) Vocabulary (Resource)
4. // ----------------------------------------------------------------------------------
5. // Version                   : 0.9
6. // Authors                   : Daniel Westphal (dawe@gmx.de)
7. //
8. // Description               : Wrapper, defining resources for all terms of the
9. //                               Resource Description Framework (RDF).
10. //                               For details about RDF see: http://www.w3.org/RDF/.
11. //                                Using the wrapper allows you to define all aspects of
12. //                             the vocabulary in one spot, simplifing implementation and
13. //                             maintainence.
14. // ----------------------------------------------------------------------------------
15. // History:
16. // 02-21-2003                 : Initial version
17. // ----------------------------------------------------------------------------------


21. class RDF{

23. // RDF concepts (constants are defined in constants.php)

25. function ALT()
26. {
27. return  new Resource(RDF_NAMESPACE_URI . RDF_ALT);

29. }

31. function BAG()
32. {
33. return  new Resource(RDF_NAMESPACE_URI . RDF_BAG);

35. }

37. function PROPERTY()
38. {
39. return  new Resource(RDF_NAMESPACE_URI . RDF_PROPERTY);

41. }

43. function SEQ()
44. {
45. return  new Resource(RDF_NAMESPACE_URI . RDF_SEQ);

47. }

49. function STATEMENT()
50. {
51. return  new Resource(RDF_NAMESPACE_URI . RDF_STATEMENT);

53. }

55. function RDF_LIST()
56. {
57. return  new Resource(RDF_NAMESPACE_URI . RDF_LIST);

59. }

61. function NIL()
62. {
63. return  new Resource(RDF_NAMESPACE_URI . RDF_NIL);

65. }

67. function TYPE()
68. {
69. return  new Resource(RDF_NAMESPACE_URI . RDF_TYPE);

71. }

73. function REST()
74. {
75. return  new Resource(RDF_NAMESPACE_URI . RDF_REST);

77. }

79. function FIRST()
80. {
81. return  new Resource(RDF_NAMESPACE_URI . RDF_FIRST);

83. }

85. function SUBJECT()
86. {
87. return  new Resource(RDF_NAMESPACE_URI . RDF_SUBJECT);

89. }

91. function PREDICATE()
92. {
93. return  new Resource(RDF_NAMESPACE_URI . RDF_PREDICATE);

95. }

97. function OBJECT()
98. {
99. return  new Resource(RDF_NAMESPACE_URI . RDF_OBJECT);

101. }

103. function DESCRIPTION()
104. {
105. return  new Resource(RDF_NAMESPACE_URI . RDF_DESCRIPTION);

107. }

109. function ID()
110. {
111. return  new Resource(RDF_NAMESPACE_URI . RDF_ID);

113. }

115. function ABOUT()
116. {
117. return  new Resource(RDF_NAMESPACE_URI . RDF_ABOUT);

119. }

121. function ABOUT_EACH()
122. {
123. return  new Resource(RDF_NAMESPACE_URI . RDF_ABOUT_EACH);

125. }

127. function ABOUT_EACH_PREFIX()
128. {
129. return  new Resource(RDF_NAMESPACE_URI . RDF_ABOUT_EACH_PREFIX);

131. }

133. function BAG_ID()
134. {
135. return  new Resource(RDF_NAMESPACE_URI . RDF_BAG_ID);

137. }

139. function RESOURCE()
140. {
141. return  new Resource(RDF_NAMESPACE_URI . RDF_RESOURCE);

143. }

145. function PARSE_TYPE()
146. {
147. return  new Resource(RDF_NAMESPACE_URI . RDF_PARSE_TYPE);

149. }

151. function LITERAL()
152. {
153. return  new Resource(RDF_NAMESPACE_URI . RDF_PARSE_TYPE_LITERAL);

155. }

157. function PARSE_TYPE_RESOURCE()
158. {
159. return  new Resource(RDF_NAMESPACE_URI . RDF_PARSE_TYPE_RESOURCE);

161. }

163. function LI()
164. {
165. return  new Resource(RDF_NAMESPACE_URI . RDF_LI);

167. }

169. function NODE_ID()
170. {
171. return  new Resource(RDF_NAMESPACE_URI . RDF_NODEID);

173. }

175. function DATATYPE()
176. {
177. return  new Resource(RDF_NAMESPACE_URI . RDF_DATATYPE);

179. }

181. function SEE_ALSO()
182. {
183. return  new Resource(RDF_NAMESPACE_URI . RDF_SEEALSO);
184. }
185. }


188. ?>
```

Documentation generated on Fri, 17 Dec 2004 16:17:21 +0100 by phpDocumentor 1.3.0RC3
